# Early Postoperative Complications and Surgical Anatomy After Ileostomy Reversal Among the Population of Khyber Pakhtunkhwa, Pakistan

**DOI:** 10.7759/cureus.19660

**Published:** 2021-11-17

**Authors:** Asadullah Khan, Muhammad Haris, Maaz Rehman, Muhammad Jehangir Khan, Abdullah ., Sobia Haris

**Affiliations:** 1 Department of Surgery, Muhammad College of Medicine, Peshawar, PAK; 2 Department of Anatomy, Nowshera Medical College, Nowshera, PAK; 3 Department of Surgery, Khyber Teaching Hospital, Peshawar, PAK; 4 Department of Pediatric Surgery, Nowshera Medical College, Nowshera, PAK; 5 Department of Surgery, Shaheed Farid Khan District Headquarter Hospital, Hangu, PAK; 6 Department of Medical Education, Nowshera Medical College, Nowshera, PAK

**Keywords:** surgical anatomy, ileostomy reversal, postoperative, complications, anastomosis

## Abstract

Objective

To assess the frequency of early postoperative complications and surgical anatomy after ileostomy reversal among the population of Khyber Pakhtunkhwa, Pakistan.

Materials and methods

In the current study, a total of 241 patients were assessed. Sufficient urine output, usual serum electrolytes and urea were indicators of a sufficient recovery. All patients remained in the ward for a minimum of seven days after surgery to detect early postoperative complications like surgical site infection (SSI), wound dehiscence, small bowel obstruction, and anastomotic leak.

Results

In the present study, 113 (47%) were in age 18-40 years, while 128 (53%) patients were in age 41-60 years. The mean age was 40±10.05. One hundred twenty-three (51%) were male, and 118 (49%) patients were female. One hundred seventy-one (71%) had ileostomy closure in ≤3 months, 70 (29%) had ileostomy closure in >3 months. The mean duration of closure was 03±3.70 months. One hundred and six (44%) had enteric perforation, 87 (36%) had blunt trauma, 48 (20%) had tuberculous abdomen. Moreover, the frequency of early complications of ileostomy closure was analyzed as 19 (8%) had surgical site infection, 14 (6%) patients had wound dehiscence, 12 (5%) patients had small bowel obstruction, and three (1%) patients had anastomotic leakage.

Conclusions

Our study concluded that early postoperative complications and surgical anatomy after ileostomy reversal among the population of Khyber Pakhtunkhwa, Pakistan were surgical site infection (8%), wound dehiscence (6%), small bowel obstruction (5%), and anastomotic leak was (1%).

## Introduction

Colostomy reversal is performed when the disease condition for which it was created has resolved, and the patient has healthy bowel ends with no distal blockage [1]. Colostomy reversal is a frequent surgical technique with little mortality and considerable morbidity [2,3]. Wound infection, ileus, and incisional hernia or anastomotic leak are all common consequences after reversal. The surgical result for colostomy reversal in terms of mortality and morbidity varies by location, based on a variety of factors such as demographic characteristics, patient-to-patient variance, and the degree of healthcare delivery [4]. As a result, even after presenting data on Western populations and a few Asian specimens, the consequence of colostomy reversal in our people cannot be projected accurately based on those research findings.

A loop ileostomy is a surgically created intestinal stoma that is used for temporary fecal diversion. The normal intestinal path is re-established after the loop ileostomy is closed, which is usually after 90 days [5]. Wound infection/hematoma, leaking from the anastomosis following reversal, small intestinal blockage at the site of ileostomy closure, iatrogenic bowel injuries, local abscess, and post reversal peristomal dermatitis are all reported postoperative issues linked with stoma closure [6,7]. The reversal has been linked to a variety of morbidity rates, according to different authors [8-10]. Ileostomy reversal is typically linked with a low rate of morbidity and death. Furthermore, ileostomy reversal may lead to complications that necessitate reoperation, with severe complications [11]. In another study, 45.9% developed some type of complication. Intestinal obstruction (32.6%), diarrhea (6%), surgical wound infection (6%), enterocutaneous fistula (4.5%), rectorrhagia (3.4%), and anastomotic leak (1.12%) were most common consequences. The average length of stay for patients was 7.54 (2-23) days [6]. Another study shows the overall complications occurred in 21.5%, including 4.8% of patients who have a serious complication; within 30 days, there was no mortality [12]. In another study, in all, 46 complications were documented in 28 individuals, resulting in a 20.3 percent overall complication rate. The incidence of anastomotic leakage was 4.3%, and the rate of reoperation was 8% [4].

The above literature suggested a variation in the complication rate from one center to another and moreover, an ileostomy is a commonly performed procedure in our setup, and studies on the outcome of its closure are rarely conducted. As no study on this issue has been done in the Pakistani population in the previous five years, it will provide us with the most recent and up-to-date facts and figures regarding the incidence of early postoperative complications and surgical anatomy of ileostomy reversal. Thus, this study was aimed to assess the early postoperative complications and surgical anatomy after ileostomy reversal among the population of Khyber Pakhtunkhwa, Pakistan.

## Materials and methods

Study design and setting

This was a descriptive cross-sectional study conducted at the Department of General Surgery at North West General Hospital, Peshawar, Pakistan.

Study duration, sample size, and sampling technique

After obtaining the ethical approval from the Institutional Ethical Review Board (IERB) of Nowshera Medical College, this study was conducted for six months from 01/01/2021 to 30/06/2021. A total of 241 was the sample size which was calculated through WHO formula for sample size calculation by taking 6% [6], the prevalence of surgical wound infection after ileostomy closure, confidence interval is 95%, and margin of error is 5% with a p-value of ≤0.05 taken as significant. Non-probability consecutive sampling method was employed in the analysis.

Sample selection

All the patients of either gender who were between the age of 18-60 years and were scheduled for ileostomy closure irrespective of indication with the duration of ileostomy >1 month including American Society of Anesthesiologists (ASA) grade I and II were included in this study. Patients that had a history of steroid intake in the last one week, diabetes, as well as patients with congenital heart disease, and those who were deemed unfit for surgery were excluded.

Data collection

All included participants were told about the study aims, risks, and advantages. Written informed consent was acquired from all participating patients at admission time. The participants were prepared for surgery for two to three hours following arrival, and all standard examinations and laboratory investigations were completed. Intravenous fluids, intravenous antibiotics, and correction of electrolyte derangements, among other things, were administered prior to surgery. Indications of appropriate resuscitation, sufficient urine production, normal serum electrolytes, and urea were used. All the patients remained admitted in the ward for at least seven days after surgery to detect early postoperative problems such as surgical site infection, wound dehiscence, small intestinal obstruction, and anastomotic leak. Name, age, sex, indication of ileostomy, and length of ileostomy were all documented on a proforma.

Data analysis

All the data was put into the SPSS version 23 for descriptive analysis (IMB Inc., Armonk, New York). For data set such as age and ileostomy duration, mean and standard deviations were calculated, whereas for categorical data such as gender, ileostomy indication, and early postoperative problems, frequencies and percentages were calculated (surgical site infection, wound dehiscence, small bowel obstruction, anastomotic leak). To investigate if there were any impact modifiers, early postoperative problems were divided by age, gender, ileostomy indication, and ileostomy duration. A Chi-square test was used, with a p-value of ≤0.05 was considered as significant.

## Results

In the present research, the total number of study participants was 241, among which 113 (47%) were in age 18-40 years, whereas 128 (53%) patients were in age 41-60 years. The mean age was 40±10.05. There were 123 males (51%), whereas 118 (49%) patients were females. Around 171 (71%) had ileostomy closure in ≤3 months, whereas 70 (29%) had ileostomy closure in >3 months. The mean duration of closure was 03±3.70 months. Almost 106 (44%) had enteric perforation, 87 (36%) had blunt trauma, and 48 (20%) had abdominal tuberculosis. Moreover, the frequency of early post-op complications of ileostomy reversal was the following: 19 (8%) had surgical site infection, 14 (6%) patients had wound dehiscence, 12 (5%) patients had small bowel obstruction, and three (1%) patients had anastomotic leak. Stratification of early postoperative complications of ileostomy closure with respect to age, gender, and period of ileostomy and indication for ileostomy is given in Tables 1-4.

**Table 1:** Stratification of early postoperative complications with respect to age

Complications		20-30 years	31-40 years	Total	p-value
Surgical site infection	yes	9	10	19	0.9651
	No	104	118	222	
Total		113	128	241	
Wound dehiscence	yes	7	7	14	0.8100
	No	106	121	227	
Total		113	128	241	
Small bowel obstruction	yes	6	6	12	0.8246
	No	107	122	229	
Total		113	128	241	
Anastomotic leak	yes	1	2	3	0.6359
	No	112	126	238	
Total		113	128	241	

**Table 2:** Stratification of early postoperative complications with respect to gender

Complications		Male	Female	Total	p-value
Surgical site infection	yes	10	9	19	0.8848
	No	113	109	222	
Total		123	118	241	
Wound dehiscence	yes	7	7	14	0.9362
	No	116	111	227	
Total		123	118	241	
Small bowel obstruction	yes	6	6	12	0.9412
	No	117	112	229	
Total		123	118	241	
Anastomotic leak	yes	2	1	3	0.5857
	No	121	117	238	
Total		123	118	241	

**Table 3:** Stratification of early postoperative complications with respect to the duration of the ileostomy

Complications		≤ 3 Months	>3 Months	Total	p-value
Surgical site infection	yes	13	6	19	0.7999
	No	158	64	222	
Total		171	70	241	
Wound dehiscence	yes	10	4	14	0.9678
	No	161	66	227	
Total		171	70	241	
Small bowel obstruction	yes	9	3	12	0.7514
	No	162	67	229	
Total		171	70	241	
Anastomotic leak	yes	2	1	3	0.8692
	No	169	69	238	
Total		171	70	241	

**Table 4:** Stratification of early postoperative complications with respect to indication for an ileostomy

Complications		Enteric perforation	Blunt trauma	Abdominal tuberculous	Total	p-value
Surgical site infection	yes	8	7	4	19	0.9836
	No	98	80	44	222	
Total		106	87	48	241	
Wound dehiscence	yes	6	5	3	14	0.9891
	No	100	82	45	227	
Total		106	87	48	241	
Small bowel obstruction	yes	5	4	3	12	0.9021
	No	101	83	45	229	
Total		106	87	48	241	
Anastomotic leak	yes	1	1	1	3	0.8356
	No	105	86	47	238	
Total		106	87	48	241	

## Discussion

Colostomy reversal is performed when the disease condition for which it was created has resolved, and the patient has healthy bowel ends with no distal blockage [1]. Colostomy reversal is a frequent surgical technique that is known to have a low death rate and considerable complication [2]. The morbidity rate for colostomy closure was found to be 29.4% in a landmark study of 6,107 individuals (5.6-49%) [3]. Wound infection, anastomotic leak, ileus, and incisional hernia are all common consequences following reversal. The surgical result for colostomy reversal in terms of mortality and morbidity varies from place to place, based on a variety of factors such as demographic characteristics, individual-to-individual variance, and the degree of healthcare provision. In this study, 113 patients (47%) were between the ages of 18 and 40, whereas 128 patients (53%) were between the ages of 41 and 60 years. The average age was 40±10.05 years. Male patients made up 123 (51%) of the total, while female patients made up 118 (49%). Ileostomy closure was achieved in 171 (71%) of cases in less than three months, and in 70 (29%) of cases in more than three months. The average closure time was 03±3.70 months. Enteric perforation was found in 106 (44%), severe trauma was found in 87 (36%), and tuberculous abdomen was found in 48 (20%). Furthermore, 19 (8%) patients experienced surgical site infection, 14 (6%) patients had wound dehiscence, 12 (5%) patients had small intestinal obstruction, and three (1%) patients had anastomotic leak.

According to one research, the total complication rate following ileostomy closure was 29%, with a 12.5% reoperation rate [10]. Ileostomy reversal is typically linked with a low rate of morbidity and death. However, ileostomy reversal may result in problems that necessitate reoperation, with severe complications ranging from 0-9% and mild issues ranging from 4-30% [11]. In another research, 45.9% of the participants experienced some sort of problem. Intestinal obstruction (32.6%), diarrhea (6%), abdominal incision infection (6%), enterocutaneous fistula (4.5%), rectorrhagia (3.4%), and anastomotic leak (3.4%) were the most common consequences (1.12%). The average length of stay for patients was 7.54 (2-23) days [6]. In another study, overall, complications occurred in 21.5%, including 4.8% patients who experienced a major complication; there were no deaths within 30 days [12]. In another research, 46 problems were observed in 28 individuals, giving in a 20.3% overall complication rate. The incidence of anastomotic leakage was 4.3%, and the rate of reoperation was 08% [4].

Our results correlate with another study carried out by Rubio-Perez I et al. [13] with a mean age of 60.3 years and a male-to-female ratio of 58%. Rectal cancer was the most common reason for ileostomy placement (56%), and 37% had preoperative chemo-radiotherapy. The average time it took for the ileostomy to close was 10.3 months. In 40% of the patients, postoperative problems developed, with 1% of them dying. Ileus (13%) and wound infection were the most common (13%). Pseudomembranous colitis was found in 4% of the cases. Delay in ileostomy closure was linked to increased postoperative complications (p=0.041). Male patients experienced greater complications (p=0.042), with wound infections (p=0.007) being the most common (p=0.007). Pseudomembranous colitis was also linked to ileostomy closure delay (p=0.003). Postoperative ileus was strongly related to end-to-end intestinal anastomosis without resection (p=0.037). Colostomy reversal is a surgery with a high rate of morbidity but a low risk of severe consequences. Overall morbidity was 41.1% in our research, which is comparable to worldwide literature (5-40%) [14]. Wound infections were the most prevalent consequence in our research (19.8%), while it was 22% in another group.

One study revealed that secondary closure following stoma reversal had no effect on wounds infection rates [15]. The kind of colostomy, the location of the stoma, the surgical method, and the presence of a drain had no effect on the colostomy closure result. The hospital stay had been extended by two days due to complications. According to recent research, in-hospital stays range from 4.2-15.5 days. One study found that individuals with no problems spent 11.1 days in the hospital, whereas those with wound infection (15.5 days), ileus (18.5 days), and anastomotic leak spent 18.5 days (20.4 days) [16].

This study was an analysis of institutional patients in order to identify risk factors for early postoperative complications and surgical anatomy after ileostomy reversal among the population of Khyber Pakhtunkhwa, Pakistan, and improve the quality of care in the Department of Surgery at Northwest General Hospital, Peshawar, Pakistan. Therefore, limitations are all those of an observational study, and due to the small number of patients, some data may not reach statistical significance. Furthermore, closure of a protective ileostomy is a fairly common surgical procedure, it has a high rate of complications, and this must be taken into account when the indication is made.

## Conclusions

Our study concludes that early postoperative complications and surgical anatomy after ileostomy reversal among the population of Khyber Pakhtunkhwa, Pakistan were surgical site infection 8%, wound dehiscence 6%, small bowel obstruction 5%, and anastomotic leak 1%. Closure of ileostomy is associated with a significant complication rate. It may use as many resources as the primary surgery and is not a minor follow-up operation. Early postoperative complications of Ileostomy reversal are a serious complication that has a great clinical impact on patients, putting surgeons in dilemmas of prevention, diagnosis, and treatment. Current practice, however, should comprise intraoperative risk assessment and subsequent adaptation of operative technique when necessary.
